# Anti-inflammatory effects of *Capparis ecuadorica* extract in phthalic-anhydride-induced atopic dermatitis of IL-4/Luc/CNS-1 transgenic mice

**DOI:** 10.1080/13880209.2020.1856146

**Published:** 2020-12-23

**Authors:** Bo Ram Song, Su Jin Lee, Ji Eun Kim, Hyeon Jun Choi, Su Ji Bae, Yun Ju Choi, Jeong Eun Gong, Jin Kyung Noh, Hye Sung Kim, Hyun-Gu Kang, Jin Tae Hong, Dae Youn Hwang

**Affiliations:** a Department of Biomaterials Science (BK21 FOUR program), College of Natural Resources and Life Science/Life and Industry Convergence Research Institute/Laboratory Animals Resources Center, Pusan National University, Miryang, Korea; b Department of Biological Science, Universidad de Concepcion Edmundo Larenas, Concepcion, Chile; c Department of Nano Fusion Technology, Pusan National University, Miryang-si, Korea; d Laboratory of Veterinary Theriogenology, Veterinary Medical Center and College of Veterinary Medicine, Chungbuk National University, Cheongju, Korea; e College of Pharmacy, Chungbuk National University, Cheongju, Korea

**Keywords:** Inflammation, iNOS, COX-2, IgE, luciferase signal

## Abstract

**Context:**

The natural products derived from *Capparis ecuadorica* H.H. Iltis (Capparaceae) could have great potential for anti-inflammation since they inhibited the inflammatory response in lipopolysaccharide (LPS)‐stimulated RAW 264.7 cells.

**Object:**

This study investigated the anti-inflammatory effects and related mechanism of methanol extract of *C. ecuadorica* leaves (MCE) during atopic dermatitis (AD) responses.

**Materials and methods:**

Alterations in the phenotypical markers for AD, luciferase signal, iNOS‐mediated COX‐2 induction pathway, and inflammasome activation were analysed in non-Tg (n = 5) and 15% phthalic anhydride (PA) treated IL-4/Luc/CNS-1 transgenic (Tg) HR1 mice (n = 5 per group), subsequent to treatment with acetone-olive oil (AOO), vehicle (DMSO) and two dose MCE (20 and 40 mg/kg) three times a week for 4 weeks.

**Results:**

MCE treatment reduced the intracellular ROS level (48.2%), NO concentration (7.1 mmol/L) and inflammatory cytokine expressions (39.1%) in the LPS-stimulated RAW264.7 cells. A significant decrease was detected for ear thickness (16.9%), weight of lymph node (0.7 mg), IgE concentration (1.9 µg/mL), and epidermal thickness (31.8%) of the PA + MCE treated Tg mice. MCE treatment induced the decrease of luciferase signal derived from the IL-4 promoter and the recovery of the IL-4 downstream regulator cytokines. PA + MCE treated Tg mice showed decreasing infiltration of mast cells (42.5%), iNOS-mediated COX‐2 induction pathway, MAPK signalling pathway and inflammasome activation in the ear tissue.

**Conclusions:**

These findings provide the first evidence that MCE may have great potential to suppress chemical-induced skin inflammation through the suppression of IL-4 cytokine and the iNOS-mediated COX‐2 induction pathway, and activation of inflammasome.

## Introduction

Atopic dermatitis (AD) is a well-known chronic inflammatory skin disease induced by various trigger factors, including food and inhalant allergens, microbial antigens, and self‐antigens (Wollenberg and Feichtner 2013; Roesner et al. 2016). Abnormal phenotypes such as itchiness, efflorescence, inflammation, redness, and small blisters on the skin are widely detected as signs and symptoms of the disease (Hiromi et al. 2004). During AD pathogenesis, the inflammatory immune response involves regulation of the humoral and the cellular immune system through T lymphocytes, mast cells, dendritic cells, and keratinocytes (Werfel et al. 2016). Especially, immune reactions induced by Th2 cells [related with interleukin (IL)-4, Il-5, IL-13, IL-31 and CCL18 secretion] and IL-22-producing T (T22) cells (related with IL-22 and S100A secretion) have been characterized in chronic AD (Czarnowicki et al. 2014; Oliva et al. 2016), whereas manifesting immunoglobulin (Ig) E autoreactivity is determined to be engaged in the development and severity of AD (Tang et al. 2012; Cipriani et al. 2014). Among the Th2 mediators, IL-4 and IL-13 cytokines play a key role in the acute and chronic stage of AD pathogenesis. These cytokines mediate the inflammatory responses in lymphocytes, myeloid cells, and non-hematopoietic cells through regulation of numerous cytokines related to the allergic response (Junttila 2018). Therefore, the regulation of IL-4 and IL-13 cytokines is considered one of the key indicators for determining the therapeutic effects of natural products in inflammatory skin diseases.

Since recent studies have demonstrated beneficial effects in animals and cell models, various natural products have received great attention as therapeutics for AD. Fermented soy crud (Sung et al. 2013), titrated extract of *Centella asiatica* (L.) Urban (Apiaceae) (Park et al. 2017), and carnosol from *Rosmarinus officinalis* L. (Lamiaceae), rosemary (Lee et al. 2017a) were found to significantly relieve the phthalic anhydride (PA)-induced AD symptoms, including ear thickness, erythema, edoema and erosion, by regulating the inducible nitric oxide synthase (iNOS)-mediated cyclooxygenase-2 (COX-2) induction pathway, inflammatory cytokines, and immunoglobulin (Ig)E secretion. Similar improvements were observed in the PA-induced AD model treated with astaxanthin, *C. asiatica* phytosome and bee venom (Park et al. 2018a, 2018b; Lee et al. 2020). Furthermore, therapeutic effects of some natural products, such as fermented soybean products, *Liriope platyphylla* Wang et Tang (Liliaceae) extract and diosgenin, were quantified using PA-treated IL-4/luciferase (Luc)/conserved noncoding sequence-1 (CNS-1) transgenic (Tg) mice (Supplementary Figure 1(A)) (Kwak et al. 2013; Lee et al. 2014; Kim et al. 2016). However, anti-inflammatory effects and the related mechanism of *C. ecuadorica* extract in the PA-induced AD model have not been studied, although several researchers continue to identify candidates for natural drugs with high anti-inflammatory activity (Panico et al. 2005; Trombetta et al. 2005).

*C. ecuadorica* is a well-known perennial shrub containing various traditional chemical substances including keratin, camphenol, ruperin, routine, stigmasterol, camping, and tocopherol. In traditional medicine, the products of this plant were rarely used to treat certain human diseases such as rheumatism, stomach problems, headaches, and toothache (Tlili et al. 2011). However, the pharmacological efficacy of other species in the family Capparaceae have been well investigated; immune-stimulation and anticancer activity of *C. zeylanica* A.Lindstr. & K.D.Hill (Cycadaceae), *C. spinosa* L. (Capparaceae), and *C. sikkimensis* Breuning (Cerambycidae) have been evaluated in peripheral blood and tumour cells (Wu et al. 2003; Ghule et al. 2006; Arena et al. 2008), as well as antidiabetic activity of *C. decidua* Edgrew (Capparaceae) and *C. spinosa* extracts has been observed in diabetic animal models (Eddouks et al. 2004, 2005). Extracts from *C. decidua*, *C. zeylanica*, and *C. spinosa* are also known to exert antibacterial activity and antioxidant activity for various microorganisms (Bonina et al. 2002; Abdalrahman et al. 2016; Kumar et al. 2019). The lyophilized extract of *C. spinosa* displays a protective effect on proinflammatory cytokine-stimulated human chondrocyte cultures, while the caper extract shows a remarkable anti-allergic effect (Panico et al. 2005; Trombetta et al. 2005). These results, presenting the therapeutic effects of other plants in the family Capparaceae, provided the possibility that natural products of *C. ecuadorica* could have great potential for anti-inflammation. Furthermore, strong evidence for this effect was provided from RAW264.7 cells. The methanol extract of *C. ecuadorica* leaves (MCE) inhibited inflammatory response in lipopolysaccharide (LPS)‐stimulated RAW 264.7 cells through the regulation of iNOS-mediated COX-2 induced pathway (Song et al. 2020).

In this study, we evaluated the possibility of developing a new natural medicine, by examining ear thickness, IL-4 promoter derived luciferase signal, iNOS-mediated COX-2 induction pathway, inflammatory cytokine levels and inflammasome activation during the anti-inflammatory effects of MCE in a PA-induced AD model.

## Materials and methods

### Preparation and extraction of MCE

The lyophilized sample of MCE (FBM206-086) was provided from the International Biological Material Research Centre of the Korea Research Institutes of Bioscience and Biotechnology (Daejeon, Republic of Korea). Briefly, dried leaf powder of *C. ecuadorica* derived from Ecuador was mixed with methanol in a fixed liquor ratio (1:10, powder–water). The mixture was repetitively subjected to the following steps: sonication for 15 min, followed by incubation for 2 h 10 times per day for 3 days, and then filtering through a 0.4 µm pore size filter. Subsequently, this methanol extract was concentrated using a Rotary Evaporator (N = 1000SWD, EYELA, Bohemia, NY, USA), and lyophilized using a Speed Vacuum Concentrator (Modulspin 40, Biotron Co., Marysville, WA, USA). The final sample of MCE was dissolved in dimethyl sulfoxide (DMSO, Duchefa Biochemie, Haarlem, Netherlands) to appropriate concentrations for use in the experiments.

### Determination of bioactive compounds in TEE

The total phenolic contents of TEE were determined using the Folin-Ciocalteu method, as previously described (Singleton and Rossi 1965). Briefly, a mixture of MCE solution (1 mL) and Folin-Ciocalteu reagent (5 mL; Sigma-Aldrich Co., St. Louis, MO, USA) was incubated at room temperature for 5 min. This mixture was subsequently added to 15 mL 20% Na_2_CO_3_ and vortexed for 30 s, after which the absorbance was repeatedly measured at 765 nm using a VersaMax plate reader (Molecular Devices, Sunnyvale, CA, USA). A standard calibration curve was generated using different concentrations of gallic acid (Sigma-Aldrich Co.), and concentration of the total phenolic compounds in TEE was presented as the gallic acid equivalent (mg) of the extract. The total flavonoid contents in TEE were measured as previously described (Zhishen et al. 1999). Briefly, 20 µL samples of varying concentrations of TEE were mixed with 60 µL 5% NaNO_2_ and 60 µL 10% AlCl_3_ (Sigma-Aldrich Co.). Following incubation at 25 °C for 5 min, the absorbance was measured using a VersaMax plate reader (Molecular Devices). A standard calibration curve was created using different concentrations of catechin (Sigma-Aldrich Co.). The total flavonoid contents of the TEE are presented as catechin equivalents (mg) of the extract. Finally, the total condensed tannin content was determined using the Vanillin method (Price et al. 1980). The extract powder of TEE was collected after dissolving in 0.5 mL 80% methanol. This extract (100 µL) was mixed with 500 µL of mixture solution (1% vanillin/MeOH and 8% HCl/MeOH, 1:1 ratio), and then incubated at 30 °C for 20 min. The absorbance was measured at 500 nm using a VersaMax plate reader (Molecular Devices). The total condensed tannin content was calculated from a calibration curve constructed using a purified (+)-catechin hydrate standard (Sigma-Aldrich Co.).

### Cell culture

RAW264.7 cells are monocytes and macrophages derived from ascites of the Abelson murine leukaemia virus-induced tumour model. These cells were procured from the Korea Cell Line Bank (Seoul, Korea), and cultured in Dulbecco’s Modified Eagle’s (DMEM) medium (Thermo Fisher Scientific Inc., Waltham, MA, USA) supplemented with 10% foetal bovine serum (FBS, S001-01, Welgene, Gyeongsan-si, Korea), l-glutamine (2 mM, Thermo Fisher Scientific Inc.), penicillin (100 U/mL, Thermo Fisher Scientific Inc.) and streptomycin (100 µg/mL, Thermo Fisher Scientific Inc.), in a humidified incubator at 37 °C under atmosphere with 5% CO_2_.

### Nitric oxide (NO) concentration analysis in RAW264.7 cells

The NO concentration was determined using Griess reagent (1% sulphanilamide, 5% phosphoric acid, 0.1% *N*-(1-naphthyl) ethylenediamine dihydrochloride; Sigma-Aldrich Co.), as described in the previous study (Sun et al. 2003). Briefly, RAW264.7 cells in each well were treated with vehicle or two different concentrations (50 and 100 µg/mL) of MCE for 2 h, followed by exposure to lipopolysaccharide (LPS) (1 μg/mL). After incubating for 24 h, Griess reagent (100 μL) was mixed with the culture supernatant of each well, and the mixture was incubated at room temperature for 10 min. Finally, the optical density was determined using a VersaMax microplate reader (Molecular Devices) at 540 nm. The concentration of NO in culture supernatants was calculated by comparing with the standard curve of sodium nitrite (NaNO_2_).

### Detection of intracellular reactive oxygen species (ROS) level

Intracellular ROS levels were measured by staining with the cell permeant reagent, 2′,7′-dichlorofluorescein diacetate (DCF-DA) (Sigma-Aldrich Co.). On attaining 70–80% confluence, RAW264.7 cells were exposed to vehicle or two different concentrations of MCE, precultured for 2 h in a 37 °C incubator, followed by LPS stimulation (1 μg/mL) for 24 h. The cells were then incubated with 100 μM DCF-DA for 15 min at 37 °C. After washing with 1× PBS, the resultant green fluorescence was observed at 200× magnification using a fluorescent microscope (Eclipse TX100, Nikon, Tokyo, Japan). The cell morphology was also observed under a light microscope (Leica Microsystems, Heerbrugg, Switzerland) at 200× magnification.

### Reverse transcription-polymerase chain reaction (RT-PCR) analysis for cytokine expression in RAW264.7 cells

RT-PCR for cytokine expression was performed as described in a previous study (Lee et al. 2017b). On reaching 70–80% confluence, RAW264.7 cells were exposed to vehicle or two different doses of MCE, incubated for 2 h, and subsequently stimulated with 1 µg/mL LPS. After incubation for 24 h, whole cells were harvested, and the total RNA was isolated using Trizol reagent (Invitrogen, Carlsbad, CA, USA). Total complementary DNA (cDNA) against mRNA was synthesized using 200 unit of Invitrogen Superscript II reverse transcriptase (Thermo Fisher Scientific). PCR was conducted with the cDNA template (2 µL), reaction mixture, and following specific primers: Tumour necrosis factors (TNF)-α, Forward: 5′-G-CCTGT AGCCC ACGTC GTAGC-3′, Reverse: 5′-TTGAC CTCAG CGCTG ACTTG-3′; IL-1β, Forward: 5′-GCACA TCAAC AAGAG CTTCA GGCAG-3′, Reverse: 5′-GCTGC TTGTG AGGTG CTGAT GTAC-3′; IL-6, Forward: 5′-TTGGG ACTGA TGTTG TTGACA-3′, Reverse: 5′-TCATC GCTGT TGATA CAATC AGA-3′; β-actin, Forward: 5′-TGGAA TCCTG TG GCA TCCAT GAAAC-3′, Reverse: 5′-TAAAA CGCAG CTCAG TAACA GTCCG −3′. All specific genes were amplified in a Perkin-Elmer Thermal Cycler for 28–32 cycles, using the following sequence: 30 s at 94 °C (denaturation), 30 s at 62 °C (annealing), and 45 s at 72 °C (extension). Finally, the PCR products for target genes were resolved on 1–2% agarose gel, and detected by ethidium bromide (EtBr). The density of each band was quantified using a Kodak Electrophoresis Documentation and Analysis System 120 (Eastman Kodak, Rochester, NY), and is represented as relative to the β-actin level.

### Production and care of IL-4/luc/CNS-1 Tg mice

Animal experimental procedures were approved by the Institutional Animal Care and Use Committee (IACUC) at Pusan National University (PNU-2018-1964). The mice were handled at the Pusan National University-Laboratory Animal Resources Centre, accredited by the Korea FDA (Accredited Unit Number-000231) and AAALAC International (Accredited Unit Number; 001525). All mice were housed under specified pathogen-free (SPF) state and a strict light cycle (lights on at 08:00 h and off at 20:00 h) and were provided *ad libitum* access to standard irradiated chow diet (Samtako, Osan, Korea).

Adult IL-4/Luc/CNS-1 Tg mice were kindly provided by the Department of Laboratory Animal Resources of the National Institute of Food and Drug Safety Evaluation (Osong, Korea) (Bae et al. 2011). IL-4/Luc/CNS-1 Tg mice and Non-Tg mice were produced by mating IL-4/Luc/CNS-1 Tg mice and HR1 mice. HR1 mice were purchased from Orient Bio Inc. (Seoungnam, Gyeonggi, Korea). Tg mice were genomically identified from the DNA extracted from tails of 4-week-old founder mice, through a DNA-PCR analysis. The inserted genes were amplified using two primer sets: sense, 5′-GAA TGT ACC AGG AGC CAT ATC-3′ and anti-sense, 5′-CTC AGT ACT ACG AGT AAT CCA-3′. Briefly, 10 pmole of sense and anti-sense primers were added, and the reaction mixture was subjected to 25 cycles of amplification conducted in a thermal cycler (Perkin-Elmer, Waltham, MA, USA) under the following conditions: 30 s at 94 °C, 30 s at 62 °C, and 45 s at 72 °C. After amplification, the product (467 bp) levels were quantified by subjecting to 1% agarose gel electrophoresis, and the band patterns were detected using Kodak Electrophoresis Documentation (Eastman Kodak) (Supplementary Figure 1(B)).

### Design of animal experiment for MCE treatment

To evaluate the therapeutic effect of MCE, 9-week-old IL-4/Luc/CNS-1 Tg mice (n = 20) were randomly divided into two groups. In the first group (acetone-olive oil (AOO), n = 5), 100 µL of AOO was repeatedly spread on the dorsum of ears, three times a week for 4 weeks, as control. In the second group (PA, n = 15), 100 µL of 15% PA solution in AOO (4:1, v/v) was repeatedly spread on the dorsum of ears, three times a week for 4 weeks. The second group was further divided into three treatment groups: PA + vehicle, PA + low concentration of MCE (MCELo), and PA + high concentration of MCE (MCEHi). The PA + vehicle treated group received a comparable volume of olive oil + 1% DMSO daily, via oral administration; the other two treatment groups were orally administered two different concentrations of MCE (MCELo, 20 mg/kg; MCEHi, 40 mg/kg) for 4 weeks. Age-matched non-Tg mice (n = 5) were used as a second control group. After the final treatment, mice of all groups were subjected to bioluminescence imaging analysis and necropsy.

### Bioluminescence imaging analysis

*In vivo* imaging analysis was conducted to detect luciferase derived signals using an IVIS imaging system (Xenogen, Oakland, CA, USA), as previously described. Briefly, IL-4/Luc/CNS-1 Tg mice were anaesthetized with Alfaxan (Alfaxalone^®^, Jurox Pty Ltd, Australia) and administered i.p. injection of 85 mg/kg d-luciferin (Sigma-Aldrich Co.). Ten minutes after the d-luciferin injection, images of the whole body and several organs [lung, kidney, spleen, heart, submandibular lymph node (SL), mesenteric lymph node (ML), thymus and pancreas] of mice from subset groups were taken for 3 min. Subsequently, the photons emitted from specific regions and organs were quantified using the Living Image software (Xenogen). The *in vivo* luciferase activity is expressed as photons per second.

### Measurement of body weight, organ weight and ear thickness

Alterations in body weight of all mice during the experimental procedure were measured once a week for 4 weeks, using an electronic balance (Mettler Toledo, Greifensee, Switzerland). Weights of SL, spleen and thymus collected from the mice of subset groups were also measured using the same electronic balance (Mettler Toledo). Ear thickness was measured once a week for 4 weeks using a thickness gauge (Digimatic Indicator, Matusutoyo Co., Tokyo, Japan), to detect the degree of allergic skin inflammation induced by PA treatment.

### Enzyme-linked immunosorbent assay (ELISA) for IgE concentration

IgE concentration in the serum was measured with an ELISA kit (Shibayagi Inc., Gunma, Japan), according to the manufacturer’s instructions. Briefly, 50 μL serum samples and standards diluted with dilution solution were added to wells coated with antibody, and subsequently incubated for 2 h at room temperature. Wells were then washed with washing solution (50 mM Tris, 0.14 M NaCl, 0.05% Tween 20, pH 8.0) three times, followed by addition of 50 μL biotin-conjugated avidin (1,000-fold dilution) to each well and incubation for 2 h at room temperature. The wells were washed again with washing solution. Horseradish peroxidase-conjugated detection antibodies (2,000-fold dilution) were added to each well and incubated for 1 h at room temperature. An enzyme reaction was initiated by adding tetra methyl benzidine (TMB) substrate solution and incubating the plate at room temperature in the dark for 20 min. Finally, the reaction was terminated by adding acidic solution (reaction stopper, 2 M H_2_SO_4_), and the absorbance of yellow product was measured spectrophotometrically at 450 nm.

### Histopathological analysis

Ear skins were collected from IL-4/Luc/CNS-1 Tg mice, fixed in 10% formalin, embedded in paraffin wax, routinely processed, and sectioned into 4 μm thick slices. The skin sections were collected on glass slides and stained with either Haematoxylin & Eosin (Sigma-Aldrich Co.) or toluidine blue (Sigma-Aldrich Co.), after which they were examined by light microscopy for cellular morphology and the presence of edoema and inflammatory cell accumulation at 100× magnification. The thickness of the epidermis and dermis, and the number of mast cells were also assessed using the Leica Application Suite (Leica Microsystems).

### Quantitative real time-PCR (RT-qPCR) analysis for cytokine expressions in lymph node

Frozen lymph node tissue was chopped with scissors and homogenized in Trizol reagent (Invitrogen Corp, Carlsbad, CA, USA). Total RNA molecules were isolated by centrifugation at 15,000 rpm for 15 min, after which the concentration was measured by UV spectroscopy. The cDNA was synthesized by Invitrogen Superscript II reverse transcriptase (Thermo Fisher Scientific Inc.). Quantitative PCR was performed with the cDNA template (2 μL) and 2× Power SYBR Green (6 μL; Toyobo Life Science, Osaka, Japan) containing specific primers. The primer sequences for target gene expression identification were as follows: TNF-α, sense primer: 5′-CCT GTA GCC CAC GTC GTA GC-3′, anti-sense primer: 5′-TTG ACC TCA GCG CTG ACT TG-3′; IL-1β, sense primer: 5′-GCA CAT CAA CAA GAG CTT CAG GCA G-3′, anti-sense primer: 5′-GCT GCT TGT GAG GTG CTG ATG TAC-3′; IL-6, sense primer: 5′-TTG GGA CTG ATG TTG TTG ACA-3′, anti-sense primer: 5′-TCA TCG CTG TTG ATA CAA TCA GA-3′; luciferase, sense primer: 5′-CAG TCG ATG TAC ACG TTC GTC AC-3′, anti-sense primer: 5′-TAG CTG ATG TAG TCT CAG TGA GC-3′; β-actin, sense primer: 5′-TGG AAT CCT GTG GCA TCC ATG AAA C-3′, anti-sense primer: 5′-TAA AAC GCA GCT CAG TAA CAG TCC G-3′. qPCR was performed for 40 cycles using the following parameters: denaturation at 95 °C for 15 s, followed by annealing and extension at 70 °C for 60 s. Fluorescence intensity was measured at the end of the extension phase of each cycle. The threshold value for fluorescence intensities of all samples was set manually. The reaction cycle at which the PCR products exceeded this fluorescence intensity threshold during the exponential phase of PCR amplification was considered to be the threshold cycle (Ct). Expression of the target gene was quantified relative to that of the housekeeping gene β-actin, by comparing the Cts at a constant fluorescence intensity, following the Livak and Schmittgen (2001) method.

### Western blot analyses

Protein homogenates from ear tissue were prepared using a protein extraction (pro-prep) solution (iNtRON Biotechnology, Burlington, MA, USA). The total proteins thus obtained were resolved on 8–12% sodium dodecyl sulfate-polyacrylamide gel electrophoresis (SDS-PAGE) for 2 h, after which the SDS-PAGE-resolved proteins were transferred (2 h at 40 V) to nitrocellulose membranes. Individual membranes were incubated overnight at 4 °C, with the following primary antibodies: anti-iNOS (Thermo Fisher Scientific Inc.), anti-COX-2 (Cell Signalling Technology, Danvers, MA, USA), anti-ERK (Santa Cruz Biotechnology), anti-p-ERK (Santa Cruz Biotechnology), anti-JNK (Cell Signalling Technology Inc.), anti-p-JNK (Cell Signalling Technology Inc.), anti-p38 (Cell Signalling Technology Inc.), anti-p-p38 (Cell Signalling Technology Inc.), anti-ASC (Cell Signalling Technology Inc.), anti-Casp-1 (Cell Signalling Technology Inc.), cleaved Casp-1 (Cell Signalling Technology Inc.), anti-NLRP3 (Cell Signalling Technology Inc.), and anti-β-actin antibody (Sigma-Aldrich Co.). The membranes were subsequently washed with washing buffer (137 mM NaCl, 2.7 mM KCl, 10 mM Na_2_HPO_4_, and 0.05% Tween 20), followed by incubation at room temperature for 1 h with 1:1,000 diluted horseradish peroxidase (HRP)-conjugated goat anti-rabbit IgG (Invitrogen, Carlsbad, CA, USA). The membrane blots were developed using the Amersham ECL Select Western Blotting detection reagent (GE Healthcare, Little Chalfont, UK).

### Statistical analysis

Statistical analyses were performed using the SPSS software version 10.10 (SPSS, Inc. Chicago, IL, USA). One-way analysis of variance followed by Tukey’s *post hoc* test, was performed to identify significant differences between the vehicle and MCE treated groups, and between the control and LPS/PA treated groups. All values presented are means ± standard deviations. A *p* value <0.05 is considered to indicate a statistically significant difference.

## Results

### Bioactive components of MCE

**Figure 1.**
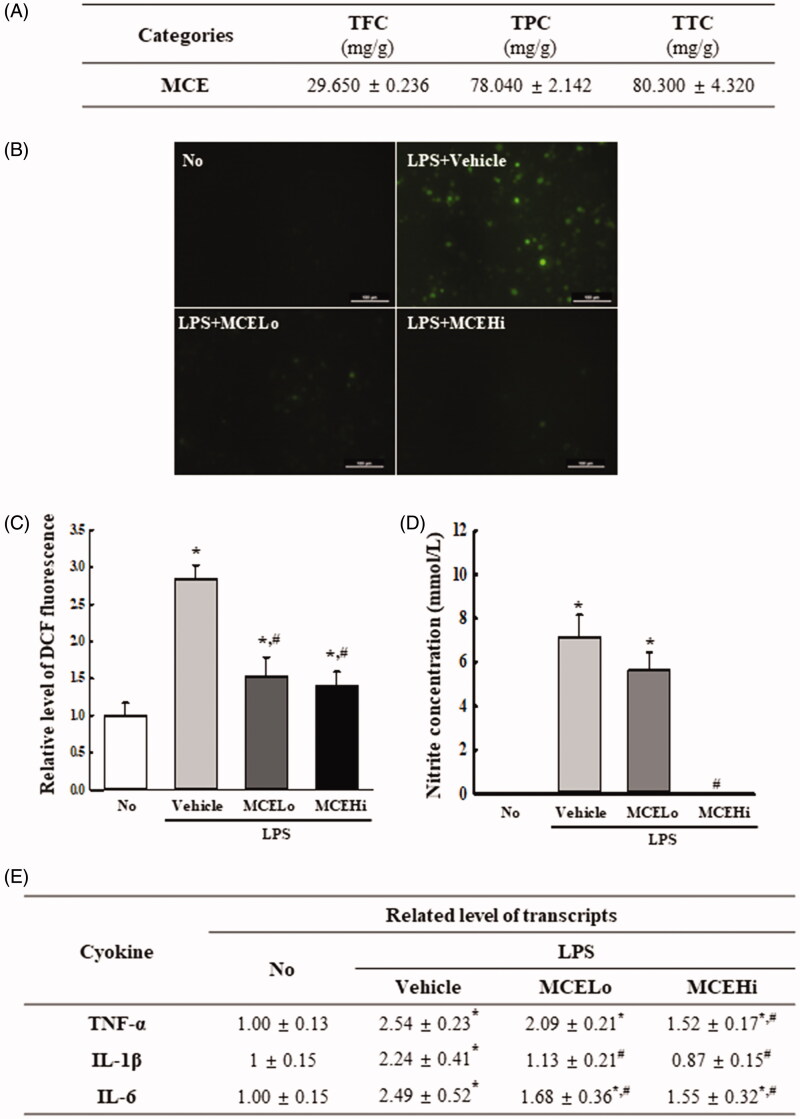
Detection of ROS, NO and cytokines in MCE + LPS treated RAW264.7 cells. (A) Phytochemical composition and component conformation in MCE. Total flavonoids, phenols and condensed tannin content were analysed in mixtures containing different concentrations of MCE. (B) After DCF‐DA treatment, the intensity of green fluorescence in RAW264.7 cells of subset groups was detected at 200× magnification using a fluorescence microscope (Eclipse TX100, Nikon, Tokyo, Japan). (C) Total number of DCF-DA stained cells were counted per specific area; relative level of stained cells in MCE treated groups are represented based on the stained cell number in No treated group. (D) RAW264.7 cells (5 × 10^5^ cells/mL) were treated with vehicle, MCELo or MCEHi in the absence or presence of LPS (1 µg/mL) for 24 h. After collecting the culture supernatants, NO concentration was measured using Griess reagent. (E) LPS-stimulated RAW264.7 cells were pre-treated with vehicle, MCELo or MCEHi for 2 h, and the expression levels of TNF-α, IL-1β and IL-6 mRNA were determined by RT-PCR. Intensity of each band was determined using an imaging densitometer, and the relative levels of the three mRNA genes were calculated based on the band intensity of β-actin as the endogenous control. Two to three wells were used for preparing DCF-DA stained cells, collecting culture supernatant, and RNA extraction; total cell number, NO concentration and mRNA expressions were measured in duplicate for each sample. Data represents the mean ± SD from duplicates. *p < 0.05 compared to the No treated group, #*p* < 0.05 compared to the LPS + vehicle treated group.

We first analysed the phytochemical composition of TEE. As showed Figure 1(A), total condensed tannin and total phenol was detected at high levels (80.3 and 78.0 mg/g) among three bioactive components. Total flavonoid contents were determined to be 29.6 mg/g, respectively. These results indicate that MCE is a tannin and phenol-enriched extract with potentially high antioxidant activity.

### Suppression of inflammatory response by MCE in LPS-stimulated RAW264.7 cells

To examine the potential of MCE as an anti-inflammatory compound in LPS-stimulated cells, alterations in the intracellular ROS levels, NO concentration, and inflammatory cytokines expression were measured in RAW264.7 cells treated with 50 and 200 μg/mL MCE in the presence of LPS (1 μg/mL) for 24 h. The intracellular ROS levels and NO concentration were remarkably increased in the vehicle + LPS treated group, when compared to the control group. However, these levels were significantly reduced with average 48.2% and 59% in cells pre-treated with MCE when compared to the vehicle + LPS treated group, although the decrease rate was varied (Figure 1(B–D)). A similar pattern was observed for the expression of inflammatory cytokines. The mRNA expression of TNF-α, IL-6, and IL-1β in LPS-stimulated RAW264.7 cells were remarkably declined with average 28.9%, 55.4% and 33.1% in all MCE treated groups (Figure 1(E)). Taken together, the above results suggest that MCE exhibits strong free radical scavenging activity, and has the potential for application as an anti-inflammatory drug having high antioxidant activity.

### Suppressive effect of MCE on the AD response induced by PA treatment in IL-4/luc/CNS-1 Tg mice

**Figure 2.**
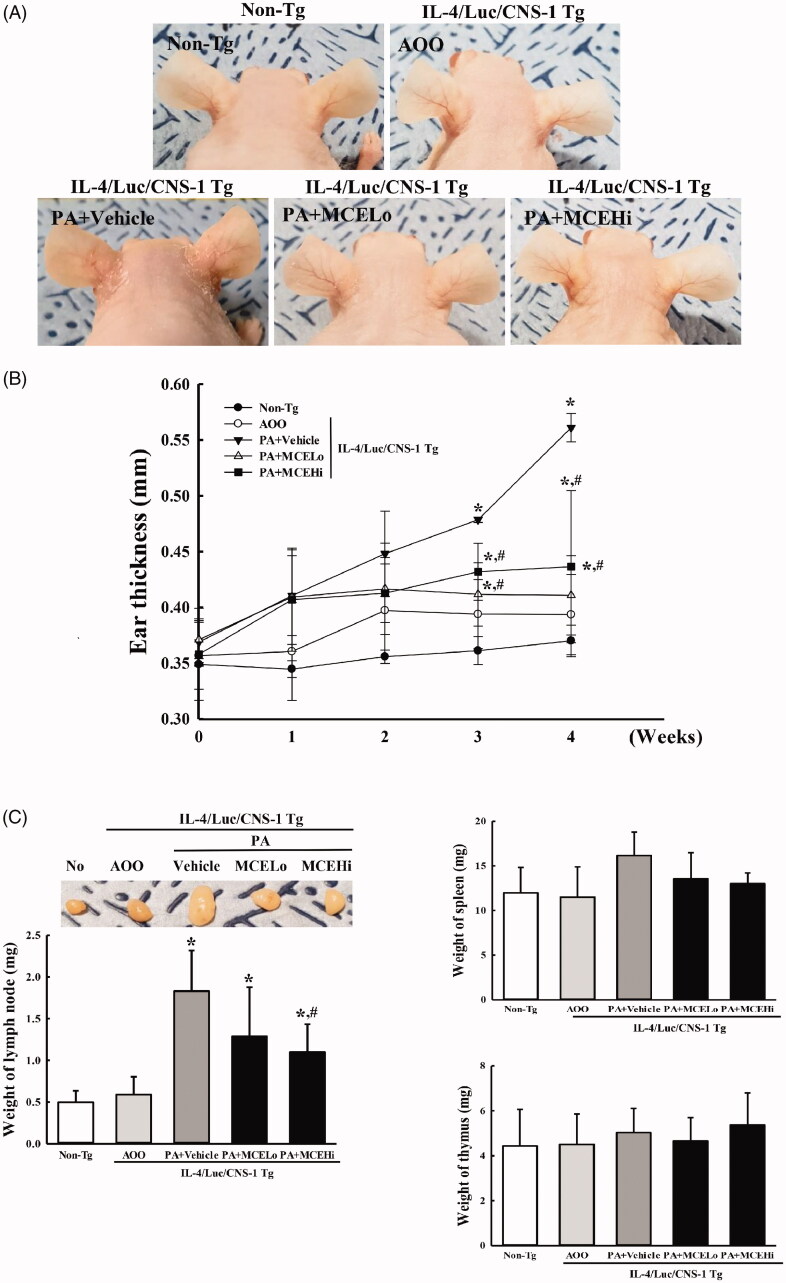
Measurement of ear morphology, ear thickness and lymph node weight after MCE treatment. (A) Ear vein and colour were analysed in photographs of IL-4/Luc/CNS-1 Tg mice treated with PA + vehicle or PA + MCE. (B) Ear thickness of mice in all five groups was measured for 4 weeks using a thickness gauge, as described in Materials and methods. (C) After final treatment, the submandibular lymph node (SL), spleen and thymus were collected from all animals in the subset groups, and their weights were measured using an electronic balance. Three to four mice per group were used in the preparation of ear image and the collection of organs, and the ear thickness and weight of organs were measured in duplicate for each sample. Data presented are the means ± SD from duplicates. *p < 0.05 compared to the AOO treated group. #*p* < 0.05 compared to the PA + vehicle treated group.

**Figure 3.**
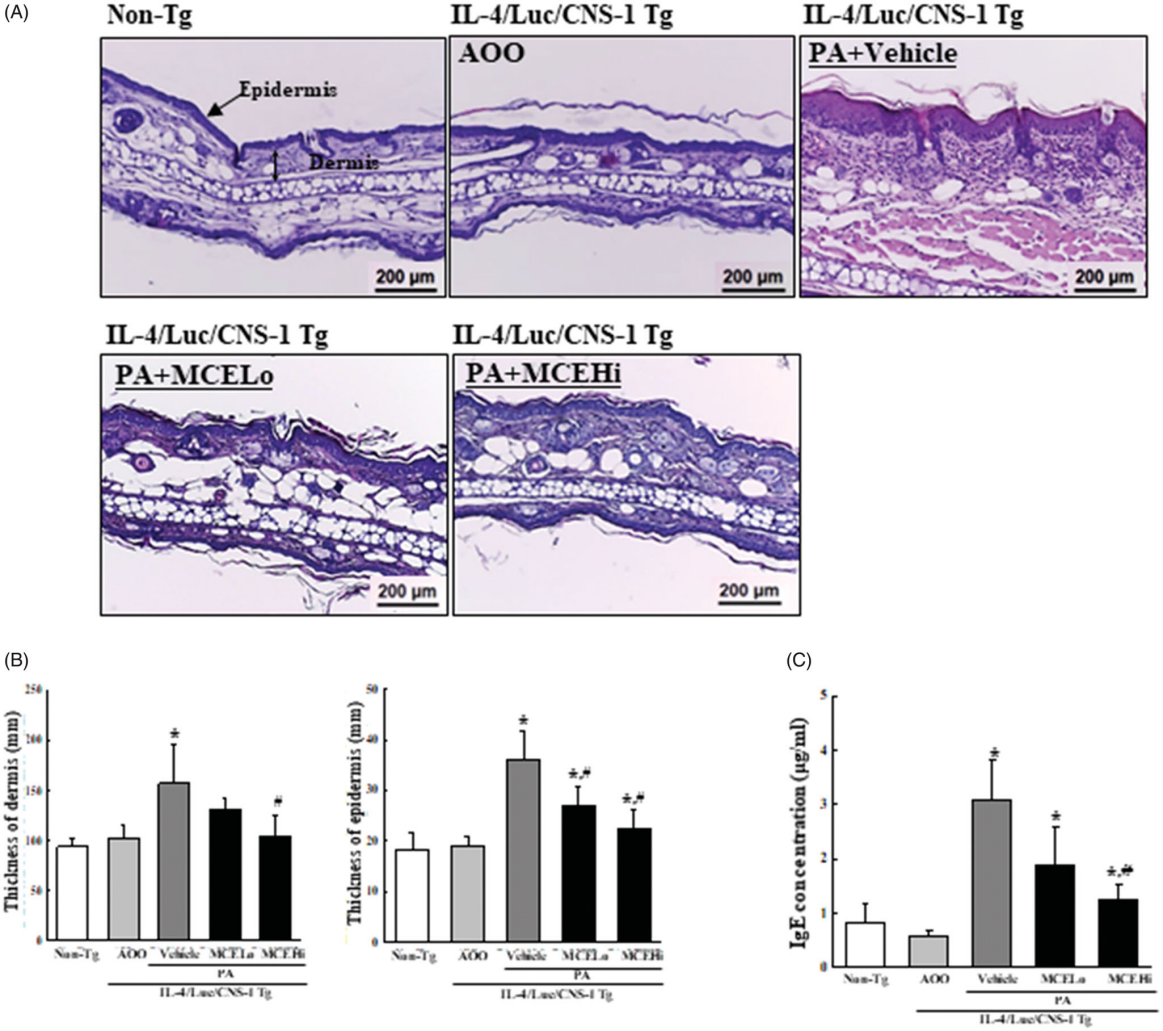
Measurement of histological structure and IgE concentration after MCE treatment. (A and B) After collecting ear tissues, histological changes were identified by staining with H&E, followed by observation at 400× magnification. (C) The concentration of IgE was quantified in serum using an enzyme linked immunosorbent assay with detectable concentration range of 10–5,000 ng/mL. Three to four mice per group were used for the collection of serum and H&E staining, and thickness measurement and ELISA was assayed in duplicate for each sample. Data shown are the means ± SD from duplicates. *p < 0.05 compared to the AOO treated group. #*p* < 0.05 compared to the PA + vehicle treated group.

To determine whether MCE administration suppresses the AD response induced by exposure to PA, alterations of general markers for AD responses, including ear morphology, ear thickness, organ weight, ear histological structure and IgE concentration, were examined in IL-4/Luc/CNS-1 Tg mice over 4 weeks. Subsequent to PA treatment, the vein outline of the mice ear became clearer or thickened, and the colour turned dark brown. However, these alterations induced by PA treatment were significantly recovered by MCE administration (Figure 2(A)). A similar recovery was detected for ear thickness, which was observed to be enhanced in the PA + vehicle treated group, relative to the AOO treated groups. However, these enhancements remarkably decreased by 10.6% and 23.2% in both PA + MCE treated groups, respectively, during the third to fourth weeks, with no significant changes in the first and second weeks (Figure 2(B)). Furthermore, the most pronounced change in weight among the three immune organs was observed in the SL. The enhanced weight of SL in the PA + vehicle treated group significantly decreased to 24.3% and 40.5% after MCELo and MCEHi treatment, respectively, although the weight of thymus and spleen showed no significant changes (Figure 2(C)). Histological analyses of the ear tissue revealed that PA treatment induces enhancement of the dermal (47%) and epidermal (50%) thickness in the ear tissue, as compared to the AOO treated group. However, the thickness decreased dose-dependently and significantly with 24.5% (dermis) and 31.8% (epidermis) in the PA + MCE treated groups (Figure 3(A,B)). A similar response was observed for the IgE concentration of serum, which was remarkably decreased in the PA + MCELo (40.6%) and PA + MCEHi treated groups (59.4%) (Figure 3(C)). These results suggest that MCE treatment successfully relieves the phenotypical markers for AD response induced by PA treatment in IL-4/Luc/CNS-1 Tg mice. Moreover, the results of the present study also indicate that alleviation of the ear thickness, lymph node weight and IgE concentration in the PA-induced AD model is dependent on the MCE concentration.

### Suppressive effects of MCE on PA-induced upregulation of IL-4 cytokine

**Figure 4.**
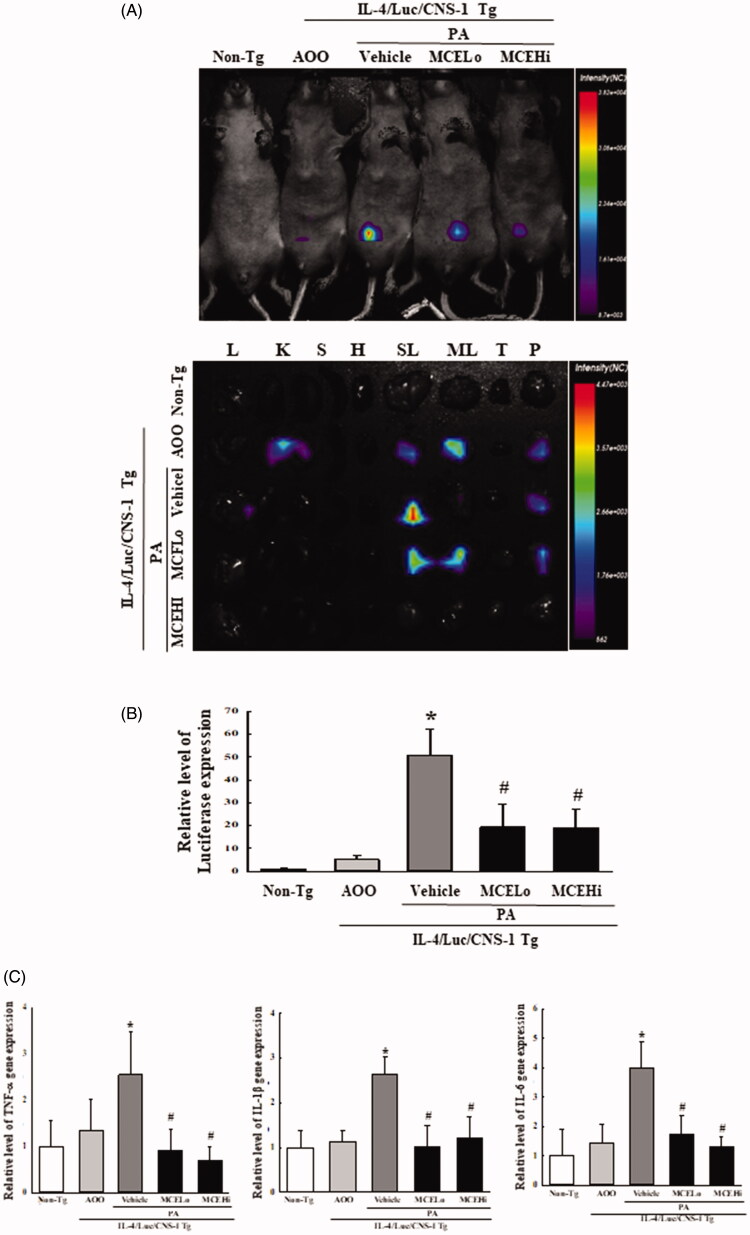
Detection of luciferase signal after MCE treatment. (A) After treatment with PA + vehicle and PA + MCE for 4 weeks, the whole body and eight organs were imaged at 24 h after the final treatment, using the Living Image software. The colour overlay in the image represents the photons per second emitted from the organs in accordance with the pseudocolor scale shown next to the image. In this image, red indicates the highest number of photons per second, while blue indicates the lowest. Abbreviations: L, lung; K, kidney; S, spleen; H, heart; SL, submandibular lymph node; ML, mesenteric lymph node; T, thymus; P, pancreas. (B) Luciferase gene of the mRNA expression was measured in the SL of PA + vehicle and PA + MCE Tg mice using RT-qPCR analyses. Three to four mice per group were used for sample preparations, and luciferase signal was assayed in duplicate. (C) The levels of TNF-α, IL-6 and IL-1α transcripts were detected in the total mRNA of SL by quantitative real time-PCR (RT-qPCR) analyses using specific primers. Three to four mice per group were used for the preparation of total RNAs, and RT-qPCR analyses were assayed in duplicate for each sample. Data shown are the means ± SD from duplicates. *p < 0.05 compared to the AOO treated group. #*p* < 0.05 compared to the PA + vehicle treated group.

Human IL-4/Luc reporter system was first applied for the production of IL-4/Luc/CNS-1 Tg mice to evaluate a respiratory sensitizer, vaccine additives, and crude extracts of natural allergens (Bae et al. 2011). We examined whether the suppressive effects of MCE on the AD response induced by PA treatment are accompanied with regulation of the IL-4 cytokine. To achieve this, luciferase signals derived from the whole body and eight organs were detected after PA + vehicle or PA + MCE treatments, using the Living Image software. High levels of luciferase signals were present only in the abdominal region of IL-4/Luc/CNS-1 Tg mice treated with PA + vehicle, while no significant signals were detected in the AOO treated group. However, the luciferase signal was remarkably decreased in the PA + MCELo and PA + MCEHi treated groups, compared to the PA + vehicle treated group. Similar suppression patterns were detected in the lung, SL and pancreas of IL-4/Luc/CNS-1 Tg mice treated with PA. The signal was dramatically decreased in the above three organs of the PA + MCELo or PA + MCEHi treated IL-4/Luc/CNS-1 Tg mice, with almost complete disappearance in the lung, SL and pancreas of the PA + MCEHi treated group (Figure 4(A)). We further measured the transcript levels of the Luc gene in SL, to verify whether the luciferase signal reflects the expression of the Luc gene. The transcript level of the luciferase gene in SL was remarkably decreased with 60.8% and 62.7% in both PA + MCE treated groups, although there was no dose-dependent response (Figure 4(B)). In addition, a similar regulation pattern was observed in the expressions of pro-inflammatory cytokines. Exposure to MCE resulted in decreased TNF-α (68.3%), IL-1β (58.9%) and IL-6 (62.5%) gene expression with complete recovery of transcripts, as compared to the AOO treated group (Figure 4(C)). Taken together, these results indicate that MCE treatment suppresses the expression of IL-4 cytokine luciferase in the lung, SL and pancreas in IL-4/Luc/CNS-1 Tg mice with PA-induced AD. Moreover, these effects are accompanied with suppression of pro-inflammatory cytokines.

### Suppressive effect of MCE on the infiltration of mast cells

**Figure 5.**
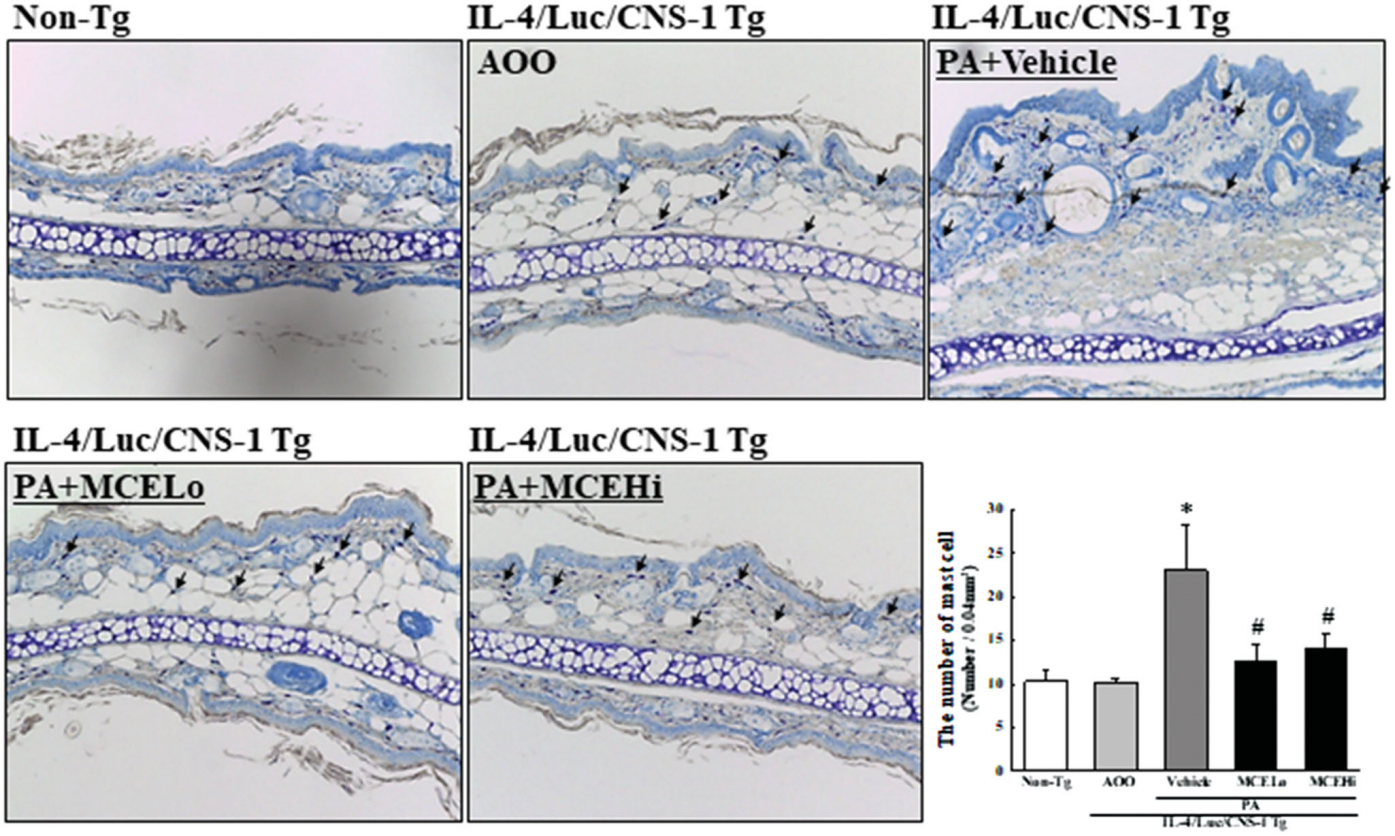
Detection of mast cell infiltration after MCE treatment. Infiltration of mast cells in the slide sections of ear tissue was identified by staining with toluidine blue, followed by observation at 400× magnification. Arrows indicate the infiltrated mast cells in the dermis of the ear tissue. Three to four mice per group were used in the preparation of toluidine blue stained sample, and the number of stained cells was counted in duplicate for each sample. Data shown are the means ± SD from duplicates. *p < 0.05 compared to the AOO treated group. #*p* < 0.05 compared to the PA + vehicle treated group.

To determine whether suppressive effects of MCE on PA-induced AD are accompanied with alterations in infiltration levels of mast cells, the number of mast cells was measured in the ear tissue of IL-4/Luc/CNS-1 Tg mice after PA + MCE treatment. PA + vehicle treated group showed high number of mast cells as compared with the AOO treated group. However, these numbers showed significant recovery (44.6% and 40.4%) in the PA + MCELo and PA + MCEHi treated groups, respectively (Figure 5). Overall, these results indicate that the effects of MCE may be tightly linked to the regulation of mast cells infiltration in the ear tissue of IL-4/Luc/CNS-1 Tg mice.

### Suppression of the iNOS‐mediated COX‐2 induction pathway of PA-induced AD by MCE treatment

**Figure 6.**
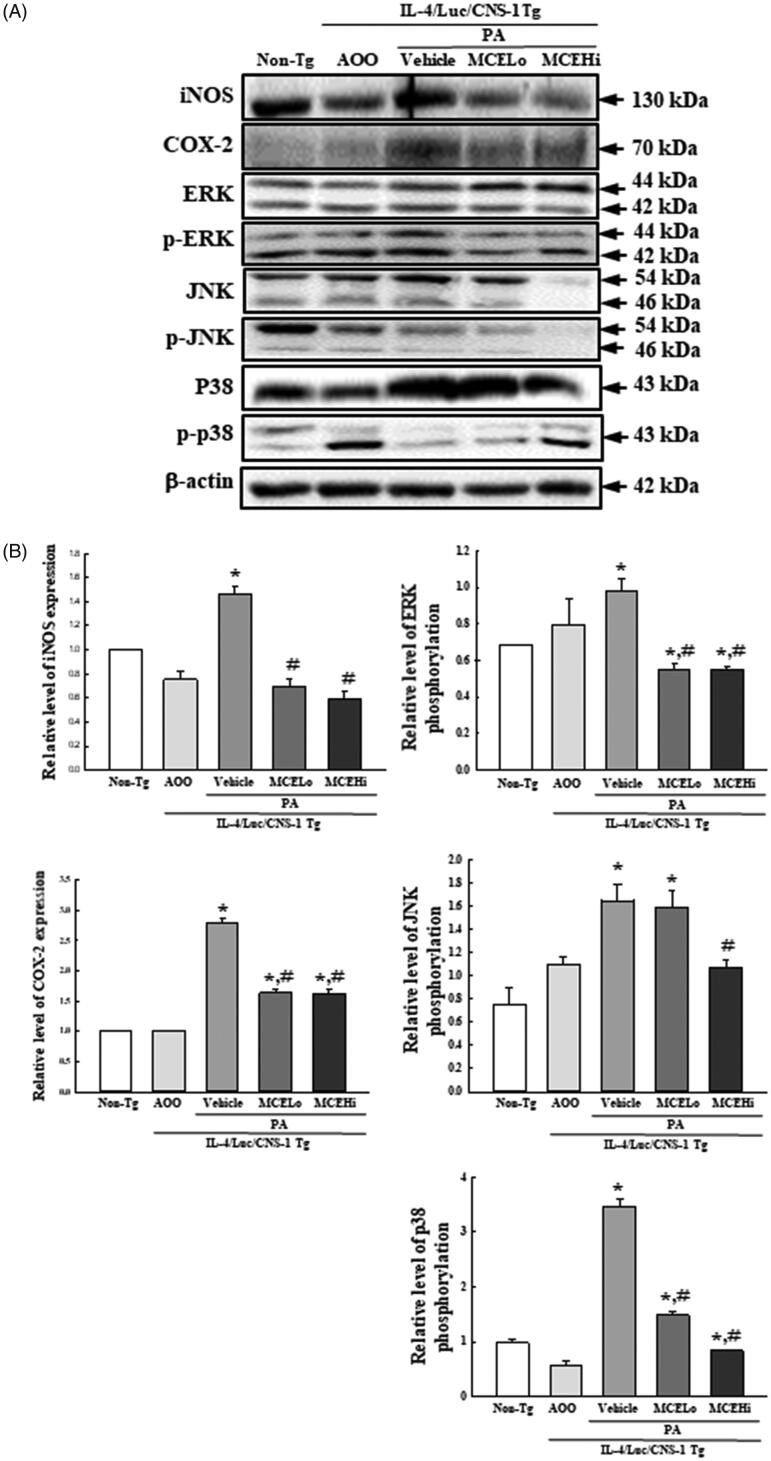
Expression of iNOS, COX-2 and MAPK members after MCE treatment. Western blot was used to detect iNOS, COX-2 and phosphorylation of ERK, JNK and p38 in the homogenates of ear tissue using specific antibodies. After determining the intensity of each band using an imaging densitometer, the relative levels of iNOS, COX-2, ERK, p-ERK, JNK, p-JNK, p38 and p-p38 proteins were calculated based on the band intensity of β-actin protein as the endogenous control. The relative phosphorylation levels of ERK, JNK and‐p38 in MCE treated cells were calculated based on the ratio of phosphorylated and non-phosphorylated proteins. Three to four mice per group were used in the preparation of tissue homogenate, and Western blot analyses were assayed in duplicate for each sample. Data represents the mean ± SD of duplicates. *p < 0.05 compared to the AOO treated group. #*p* < 0.05 compared to the PA + vehicle treated group.

NO plays an important role as one of the mediators during the inflammatory response (Wallace 2005). To investigate whether suppressive effects of MCE on PA-induced AD are accompanied with alterations in iNOS‐mediated COX‐2 induction pathway, we analysed the expression levels of iNOS and COX-2 proteins in the ear tissue of PA + MCE treated mice. As presented in Figure 6, similar patterns of iNOS and COX-2 expression were observed in the subset groups. These levels were higher in the PA + vehicle treated group, when compared to the AOO treated group and No treated group. However, the expression levels of iNOS and COX-2 proteins were remarkably and dose-dependently decreased in the PA + MCE treated group, as compared to the PA + vehicle treated group. Also, the recovery of both protein expressions was accompanied with the recovery of c-JUN N-terminal kinase (JNK), extracellular signal-regulated kinase (ERK) and p38 phosphorylation in the same tissues. Thus, we deduce that treatment of MCE suppresses the inflammatory response in the ear tissue through regulation of the iNOS‐mediated COX‐2 induction pathway and MAPK signalling pathway.

### Suppression of inflammasome activation by MCE treatment

**Figure 7.**
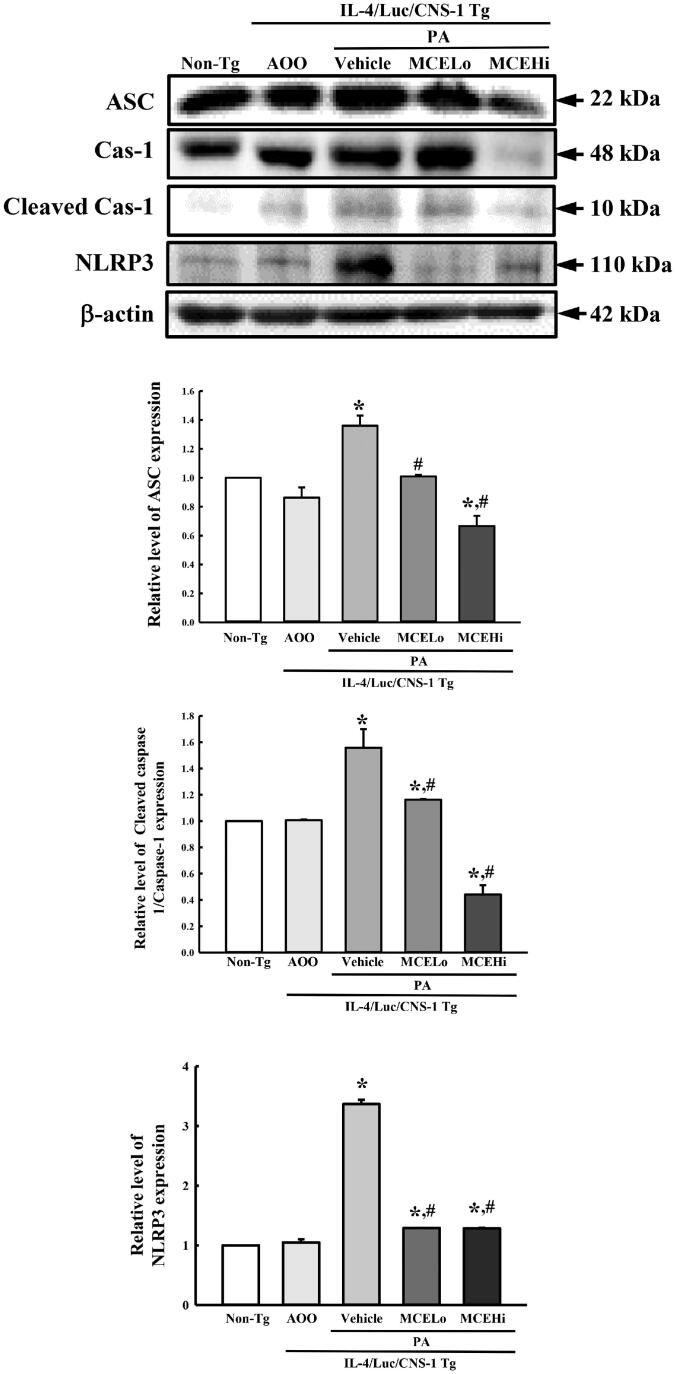
Expression of ASC, Casp-1 and NLRP3 protein after MCE treatment. Western blot was performed to detect ASC, Casp-1 and NLRP3 proteins in the homogenates of ear tissue, using specific antibodies. After determining the intensity of each band using an imaging densitometer, the relative levels of ASC, Casp-1 and NLRP3 proteins were calculated based on the band intensity of β-actin protein as the endogenous control. Three to four mice per group were used in the preparation of tissue homogenate, and Western blot analyses were assayed in duplicate for each sample. Data represents the mean ± SD of duplicates. *p < 0.05 compared to the AOO treated group. #*p* < 0.05 compared to the PA + vehicle treated group.

Inflammasome is a multiprotein intracellular complex that detects pathogenic microorganisms and sterile stressors. They are activated as a key functional mediator during innate immunity (Wang et al. 2020). We investigated whether suppressive effects of MCE on PA-induced AD are accompanied with alterations in inflammasome activation. To achieve this, the expression levels of apoptosis-associated speck-like protein containing a CARD (ASC), caspase-1 (Casp-1) and NLR family pyrin domain containing 3 (NLRP3) were measured in the ear tissue of PA + MCE treated groups. The levels of all three proteins were higher in the PA + vehicle treated group as compared to the PA + AOO treated group. However, these levels were significantly reduced with 39.9% (ASC), 49.4% (Casp-1) and 77% (NLRP3) after MCE treatment, although their rate was varied (Figure 7). These results indicate that the anti-inflammatory effects of MCE in AD may be tightly associated with the suppression of inflammasome activation.

## Discussion

Anti-inflammatory effects of natural products on AD has traditionally been evaluated using various parameters, including ear thickness, IgE concentration, lymph node weight, epidermal thickness and cytokine levels, in cell lines and animals treated with allergens (Yun et al. 2014; Man et al. 2018). However, in this field of research, the application of new animal models and novel parameters for pharmaceutical efficacy has only recently been attempted. Also, novel natural products are continually reported to be important sources for the treatment of anti-inflammatory related diseases, although scientific evidence for their therapeutic effects are insufficient. As part of such research, we investigated the anti-inflammatory effects of MCE in the PA-induced AD model. To achieve this, the effects of MCE on the phenotypical markers, iNOS-mediated COX-2 induction pathway, and inflammasome activation were analysed in IL-4/Luc/CNS-1 Tg mice treated with PA + MCE. Our results provide the first evidence that MCE alleviates the PA-induced skin inflammation in IL-4/Luc/CNS-1 Tg mice. We propose that these effects of MCE are tightly correlated with regulation of the iNOS-mediated COX-2 induction pathway and inflammasome activation.

IL-4/Luc/CNS-1 Tg mice were first produced by our research team as animal models for the prediction and evaluation of human immune response to various allergens (Bae et al. 2011). In previous studies, this mouse model was applied for quantitatively evaluating the therapeutic effects of natural products on atopic dermatitis. The luciferase signal derived from the Luc vector system was significantly decreased in the entire body and in several organs after treatment with aqueous extracts of *L. platyphylla* (AEtLP), fermented soybean products, diosgenin, and acetate extract of *Asparagus cochinchinensis* Merr. (Asparagaceae) (Kwak et al. 2013; Lee et al. 2014; Kim et al. 2016; Sung et al. 2016). Also, the IL-4/Luc/CNS-1 Tg mice were used to detect allergic responses induced by repeated dermal exposure to low doses of formaldehyde (Kwak et al. 2014). Based on the above previous results, we selected the IL-4/Luc/CNS-1 Tg mice for the current study, to investigate the anti-inflammatory effects of MCE on PA-induced AD. Results obtained in the present study are very similar to previous studies that evaluated the therapeutic effects of natural products using the IL-4/Luc/CNS-1 Tg mice. Therefore, our results provide additional evidence that IL-4/Luc/CNS-1 Tg mice are valuable for evaluating natural products exerting high anti-inflammatory activity.

Meanwhile, the concentrations of MCE used this study were determined based on the results of several *in vitro* and *in vivo* previous studies. MCE effectively inhibited inflammatory responses at 50, 100, and 200 µg/mL in LPS‐stimulated RAW 264.7 cells without any significant toxicity (Song et al. 2020). Most of other natural products including *A. cochinchinensis* mixture, pomegranate flower, *Rhodomyrtus tomentosa*, and *Cinnamomum camphora* were treated in LPS‐stimulated RAW 264.7 cells at 10 to 400 µg/mL to evaluate their anti-inflammatory effect (Lee et al. 2006; Jeong et al. 2013; Lee et al. 2017b; Xu et al. 2017). Furthermore, in previous studies using IL-4/Luc/CNS-1Tg mice, many natural products and chemical compound including *L. platyphylla*, red *L. platyphylla*, diosgenin and Cheonggukjang were administrated at 25 and 50 mg/kg for 4 weeks, while only *A. cochinchinensis* were treated at 200 and 400 mg/kg for 2 weeks (Kwak et al. 2013, 2017; Lee et al. 2014; Kim et al. 2016; Sung et al. 2016). Therefore, concentrations of less than 50 mg/kg were considered as primary concentrations for administering MCE. But, the optimal dose of MCE was determined at 40 mg/kg for administrating IL-4/Luc/CNS-1 Tg mice because of their maximum saturation concentration.

Until now, there are no reports for MCE ingredients although the correlation between ingredients of natural products and therapeutic effects is considered as one of important factors in most efficacy studies. In this study, we firstly analysed the ingredients of MCE. As shown Figure 1(A), MCE contained 80.3 mg/kg of total tannin content, 78 mg/kg total phenol content and 29.6 mg/kg of total flavonoid content. These secondary metabolites including phenolic compounds, flavonoids, alkaloids, and tannins are widely distributed in roots, stem and leaves of many plants (Medini et al. 2014; Rebaya et al. 2014; Rahimi Khoigani et al. 2017). They play an important role on various biological effects such as antioxidant, free radical scavenging activity and anti-inflammatory response (Miller 1996). However, the ingredients for leaves of other species in the same family were partly analysed. Quercetin triglycoside, 3-*O*-[6_n_-α-l-rhamnosyl-6″-β-d-glycosyl]-β-d-glucoside, and tocopherol were identified in leaves mixtures of *C. spinosa*, while four types of glucosinolate were detected in leaves of *C. flexuosa* (Sharaf et al. 2000; Tlili et al. 2009). Also, several types of flavonoid glycosides and flavonol aglycones were identified in the leaves and stems of *C. cartilogenia*, *C. deciduas* and *C. spinosa* (Sharaf et al. 1997; Pelotto and Del Pero Martínez 1998). Therefore, further studies required to identify the ingredients that have anti-inflammatory activity from leaves of *C. ecuadorica*.

Results of the current study revealed that MCE treatment for 4 weeks induces recovery of the iNOS-mediated COX-2 induction pathway in PA-induced AD of IL-4/Luc/CNS-1 Tg mice. The enhanced levels of iNOS and COX-2 expressions were significantly decreased after treatment with low and high concentrations of MCE (Figure 7). These results are very similar with some previous studies which reported inhibition of the iNOS-mediated COX-2 induction pathway in AD models after exposure to several natural products. The expression levels of iNOS and COX-2 mRNA and proteins were remarkably decreased after treatment with oregonin from *Alnus japonica* Steud (Betulaceae) (Choi et al. 2010), taxifolin 3-*O*-β-d-glucopyranoside from *Rhododendron mucronulatum* Turez (Ericaceae) (Ahn et al. 2010), and *A. japonica* extract (Choi et al. 2011), in diphenylcyclopropenone (DPCP)-treated NC/Nga mice. Similar effects were observed in mite cream-exposed NC/Nga mice treated with two condensed tannins from the root of *R. multiflora* Thunberg (Park et al. 2011) and *Rose multiflora* root extracts (Park et al. 2014). Hence, the iNOS-mediated COX-2 induction pathway is considered an important factor for evaluating the anti-inflammatory potential of natural products in AD animal models.

Inflammasome is a well-known multiprotein cytosolic complex and intracellular sensor that detects a broad range of microorganisms during response of the innate immune system (Schroder and Tschopp 2010). This complex is activated by a group of pattern recognition receptors (PRRs) and damage-associated molecular patterns (DAMPs) (Jo et al. 2016). Inflammasomes contain a nucleotide-binding oligomerization domain-like receptor (NLR) protein as the main component, and is classified into four types: the NLRP1/NALP1b inflammasome (Boyden and Dietrich 2006), the NLRC4/IPAF inflammasome (Miao et al. 2006; Zhao et al. 2011), the NLRP3/NALP3 inflammasome (Martinon et al. 2006), and the AIM2 (absent in melanoma 2) containing inflammasome (Fernandes-Alnemri et al. 2009; Hornung et al. 2009). However, inflammasomes have not received great attention for the pathogenesis of AD until recently, although abnormal regulation of inflammasome was observed in various auto-inflammatory syndromes and autoimmune diseases (Franchi et al. 2012). Only a few studies have investigated the role of inflammasome activation in AD pathogenesis. The expression of several inflammasome components was increased in the 2,4-dinitrochlorobenzene (DNCB)-induced AD animal model, along with enhancement of ear thickness, lymph nodes weight and IgE concentration (Jang et al. 2018). Also, NLRP3 inflammasomes were activated in the *in vitro* cell model of AD induced by LPS and house dust mite (Dai et al. 2011; Wang and Ma 2019). However, most studies correlating skin disease and inflammasome were focussed only on psoriasis (Hiruma et al. 2018; Zhang et al. 2018). In the current study, we investigated the role of MCE on the molecular mechanism of inflammasome activation in the PA-induced AD model. Enhanced levels of NLRP3, ASC and Casp-1 expressions was recovered in the IL-4/Luc/CNS-1 Tg mice after PA + MCE treatment. These results are very similar to previous studies, although the methods used to induce the atopic disease were different. We believe that our results provide the first evidence that the anti-inflammatory effect of MCE is probably associated with inhibition of inflammasome activation in the PA-induced AD model. However, further studies are required to advance our understanding of the impending effects of a single compound, as well as the pharmaceutical mechanisms responsible for these effects.

## Conclusions

Taken together, results of the present study demonstrate that MCE exerts its anti-inflammatory activity by successfully suppressing the increase of ear thickness and lymph node weight, mast cells infiltration, enhancement of cytokine expression, and elevation of IgE concentration, in PA-induced IL-4/Luc/CNS-1 Tg mice. In addition, the anti-inflammatory effects of MCE were detected using the luciferase signal derived from the IL-4 report system, as well as the suppression of iNOS-mediated COX-2 induction pathway and inflammasome activation. The regulatory effects of MCE on the NO production and inflammasome activation during suppression of AD indicate the potential of MCE as an anti-atopic dermatitis drug that results in normal skin barrier formation.

## Supplementary Material

Supplemental Material
