# Supplementary figures and images for: Anti-inflammatory effects of Capparis ecuadorica extract in phthalic-anhydride-induced atopic dermatitis of IL-4/Luc/CNS-1 transgenic mice
Source: Pharm Biol. 2020 Dec 23;58(1):1272–85. doi: 10.1080/13880209.2020.1856146 (PMC7782699; doi:10.1080/13880209.2020.1856146)

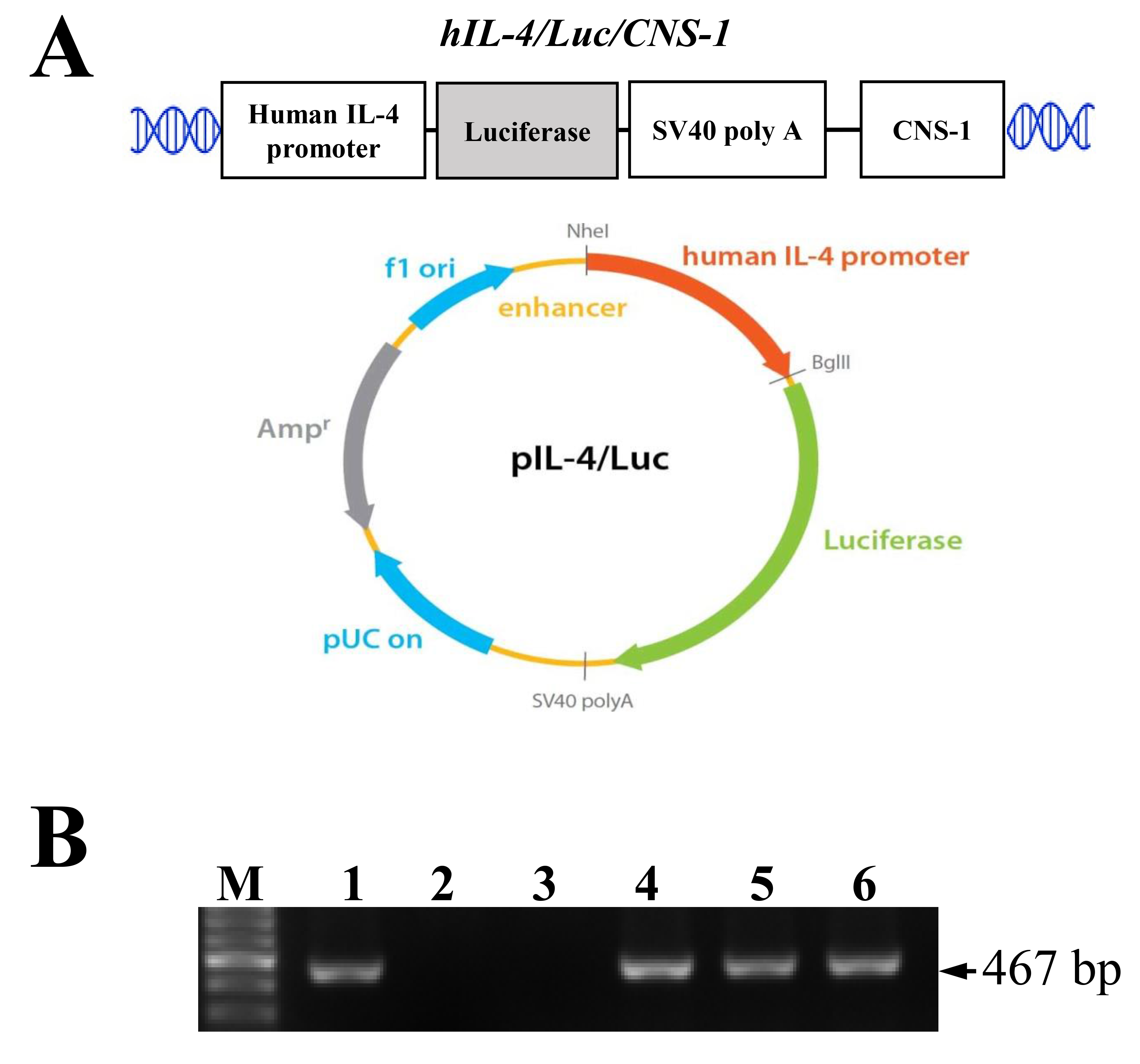

Supplement: Supplemental Material [file IPHB_A_1856146_SM9672.tif]
